# Remifentanil but not sufentanil induces cardioprotection in human ischemic heart muscle in vitro

**DOI:** 10.1186/s40360-023-00660-3

**Published:** 2023-04-20

**Authors:** Marcin Kunecki, Tomasz Oleksy, Jan Martynów, Michalina Zygmunt, Marek Deja, Tomasz Kargul, Jolanta Biernat, Piotr Podolec, Krzysztof S. Gołba, Wojciech Płazak

**Affiliations:** 1 Department of Electrocardiology and Heart Failure, Medical University of Silesia, Katowice, Poland; 2 Department of Anestesiology and Intensive Care, John Paul 2nd Hospital, Jagiellonian University Medical College, Krakow, Poland; 3 Department of Cardiac and Vascular Diseases, John Paul 2nd Hospital, Jagiellonian University Medical College, Krakow, Poland; 4 Department of Cardiosurgery, Medical University of Silesia, Katowice, Poland

**Keywords:** Ischemia–reperfusion injury; remifentanil, Sufentanil, Cardioprotection

## Abstract

**Background:**

Previous studies on animal models have suggested that δ-opioid receptor (OR) signaling is the primary pathway responsible for opioids' cardioprotective effect. We hypothesize that the μ-OR's activation protects the human heart muscle.

**Methods:**

We performed the experiments on muscular trabeculae obtained from the right atrial appendages of 104 consecutive patients subjected to coronary artery bypass surgery. Two trabeculae from each patient were studied simultaneously and exposed to 60 min of hypoxia with subsequent 60 min of reoxygenation. Remifentanil (5 μM or 50 μM) or sufentanil (40 μM or 400 μM) was used from the time of reoxygenation. Trabeculae contractility was assessed as the maximal amplitude of the contraction at baseline, after 60 min of hypoxia, during reoxygenation, and after norepinephrine application.

**Results:**

During reperfusion, the application of remifentanil improved cardiomyocytes' function as compared to the control group (time from reperfusion: 15 min: 39.8% vs. 21.7%, *p* = 0.01; 30 min: 41.4% vs. 21.8%, *p* = 0.01; 60 min: 42.7% vs. 26.9%, *p* = 0.04; after norepinephrine: 64.7% vs. 43.2%, *p* = 0.03). The application of sufentanil did not influence cardiomyocyte function as can be seen when comparing the results of the experimental and control group.

**Conclusions:**

Remifentanil, but not sufentanil, induces a cardioprotective effect on human right atria muscle in in vitro conditions, manifested as the increased amplitude of their contraction during reperfusion after 60 min of ischemia.

## Introduction

Ischemic heart disease in adults remains the leading cause of morbidity and mortality. Acute coronary syndrome treatment is a percutaneous coronary intervention to restore coronary perfusion. However, the reperfusion triggers a cascade of intracellular reactions, increasing the final injury up to 50% [1, 2]. Sequences of brief periods of non-lethal ischemia and reperfusion applied before or after the coronary occlusion are evidenced to decrease ischemia–reperfusion injury (IRI) [3–5]. The main problem with finding a practical aspect of ischemic conditioning was overcome with application of the remote ischemic conditioning. However, this strategy has shown neutral results in two large scale clinical trials [6, 7] Substantial research efforts have been made to find pharmacological agents that can mimic these cardioprotective strategies since using these procedures in humans is impractical. Moreover, the results from human trials have been controversial. The mechanisms of ischemic preconditioning or postconditioning are still not fully understood. However, there is strong experimental evidence that opioids participate in the endogenous cardioprotective response to IRI [8–10].

Many research studies have shown that opioids administered at the beginning of the reperfusion may also protect heart muscles against IRI. However, the underlying mechanism has not been fully elucidated. Our previous studies on human heart tissue [2] have shown that the cardioprotective effect may be due to μ-OR rather than δ-OR activation. This data remains contrary to research performed on an animal model of IRI, which suggests the primary role of δ-OR stimulation [11–13]. Other studies with animal models reported that κ-OR but not δ-OR stimulation resulted in both infarct size limiting and an antiarrhythmic effect [14, 15].

Remifentanil is a short-acting opioid drug widely used in anesthesia because of its quick elimination and weak impact on hemodynamics. The effects of remifentanil are connected with μ-OR agonism. However, remifentanil is also a partial agonist δ-OR and NMDA receptor [16].

Sufentanil is a selective μ-OR agonist also widely used in anesthesia. It has been shown that sufentanil may be a part of the endogenous cardioprotective response to IRI and trigger intracellular enzyme cascades, ultimately leading to the closure of mitochondrial permeability transition pores (mPTP) responsible for the induction of cell damage [17, 18].

Previous studies on animal models have suggested δ-OR signaling as the primary pathway involved in the beneficial effect of opioids. We hypothesize that the μ-OR activation provides cardioprotection in human heart muscles, which may explain the protective mechanisms against IRI in humans.

The study aims to establish the possible cardioprotective effect of remifentanil and sufentanil on ischemic human heart muscle cells in vitro conditions.

## Materials and methods

Our experiments were performed using muscular trabeculae obtained from the right atrial appendages of 104 consecutive patients of Department of Cardiosurgery, Medical University of Silesia in Katowice, subjected to coronary artery bypass surgery. We presented the patients' demographic data in Table 1.

**Table 1:** Characteristics of the patients from whom myocardial fragments were taken

Number of patients	104 (100%)
Males	82 (79%)
Females	22 (21%)
Age (years)	64,8 ± 9.7
Left ventricle ejection fraction (%)	50,7 ± 8,5
Diabetes	23 (22%)
Diabetes on insulin treatment	10 (9%)
Beta-blockers	97 (93%)
Calcium channel blockers	23 (22%)
ACE inhibitors	67 (64%)
Angiotensin receptor blockers	2 (2%)
Aspirin	89 (86%)
Statins	89 (86%)

We excluded the patients diagnosed with severe valvular heart disease or significant heart failure from the study. It is possible to obtain maximum two trabeculae from human atrial appendices. To make our results the most reliable, trabeculae from each patient were used simultaneously. Fragments of the human right heart atria explanted during surgery were transported from the cardiac surgery room to the laboratory in the ice-cold Krebs–Henseleit solution ([M]: 118.0 NaCl, 24.88 NaHCO3, 1.18 KH2PO4, 1.64 MgSO4, 4.70 KCl, 1.52 CaCl2, 2.0 sodium pyruvate, 11.0 glucose; pH 7.4). Two muscular trabeculae were obtained from the right heart appendage and incubated in separate organ baths (Hugo Sachs Elektronik—HSE, Schuler Organ Bath, March-Hugstetten, Germany). Both were filled with the 37 °C Krebs–Henseleit solution. The trabeculae used in the study had a cross-sectional area below 1 mm in diameter to avoid core hypoxia. There were always studied simultaneously two trabeculae from each patient and exposed to the hypoxia protocol, including 60 min of hypoxia (incubation in the Krebs–Henseleit buffer saturated with 95% argon and 5% carbon dioxide deprived of glucose and pyruvate) with a subsequent 60 min period of reoxygenation (incubation in the Krebs–Henseleit buffer saturated with the 5% carbon dioxide and 95% oxygen). The buffer was replaced every 15 min, except for the period of hypoxia.

According to the Frank-Starling relationship, each trabecula was stretched to 90 percent of its optimal tension strength. All trabeculae were driven throughout the experiments with 1 Hz 50 ms square stimuli using a stimulator Type 215 (HSE) and platinum field electrodes. Every trabecula's contractive function was analyzed using the transducer (Type 372, HSE). The signal was amplified with a bridge amplifier (Type 336, HSE), recorded with the PowerLab/4SP system, and calculated using chart software (ADInstruments, Chalgrove, UK). Each experimental protocol ended with the application of 10 μM of norepinephrine to assess trabecula viability.

Control trabeculae were subjected only to the hypoxia protocol. Remifentanil (5 μM or 50 μM) or sufentanil (40 μM or 400 μM) were used from the beginning of the reoxygenation period. Both remifentanil and sufentanil were used every five min during the experiment. To make our results the most reliable, trabeculae from each patient were used simultaneously. We can directly assess the effect of drug comparing with control in equal tissue. This study construction can avoid the influence of disruptive factors like individual variability. Trabecula contractility was assessed as the contraction's maximal amplitude. Measurements were obtained at baseline, after 60 min of hypoxia, during reoxygenation (at the fifth, 10th, 15th, 30th, 45th, and 60th min), and after norepinephrine application. The data for reperfusion's onset after 60 min of hypoxia ("0 min") were not analyzed due to the many artifacts caused by mechanical influence during the experiment (including changes of gas supply and opioid application).

We presented the results as percentages of the values of contraction amplitudes obtained at the beginning of the experimental protocol. At the onset of experiment, after trabeculae stability obtained in normoxic conditions, measurements were done as a baseline (100% of values). The following measurements were a percentages of the values of contraction amplitudes obtained at the beginning of the experimental protocol. Norepinephrine was used in the end of each experiment to check the heart muscle viability and to exclude the effect of myocardial stunning. All results are presented as a mean with a standard error of the mean (SEM). Two-way analysis of variance (ANOVA) and the Holm-Sidack test were used to compare the values from the fifth to the 60th min of reoxygenation; the p values < 0.05 were considered statistically significant. Statistical analysis was performed using SigmaPlot software v. 10.0.1.2 (Systat Software Inc., San Jose, USA).

## Results

**Fig. 1:**
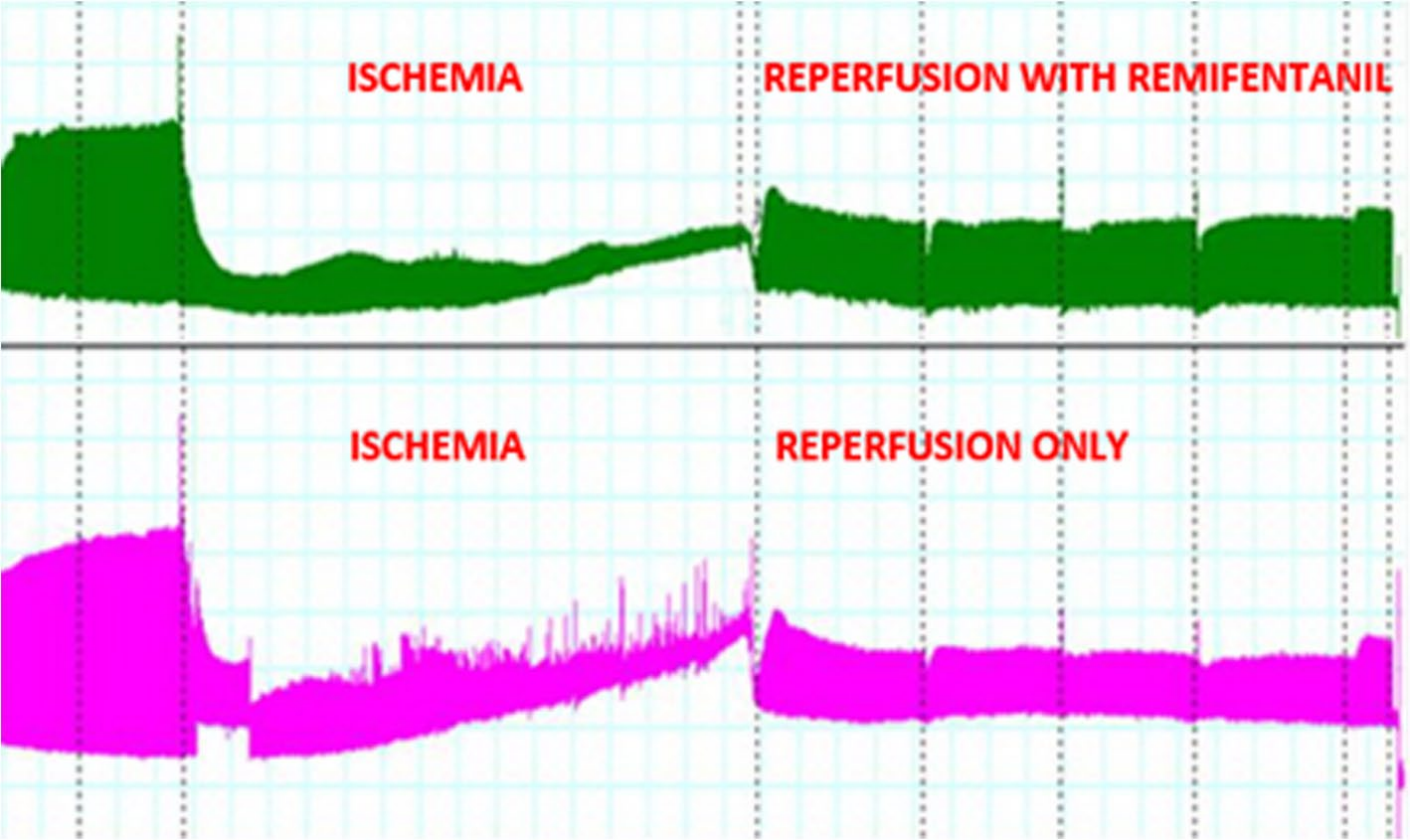
Effects of hypoxia and reperfusion on the contractile function of cardiomyocytes. An example of the recording obtained during the experiment. The trabeculae's contraction amplitudes are shown for remifentanil (above) and the control (below). A profound decrease in trabeculae contraction results from ischemia. At the end of 60 min of ischemia, oxygen is again applied (reperfusion), and opioid is added to the study probe. Higher contraction amplitudes are noted for the remifentanil probe

Figure 1 shows an example of the recording obtained during the experiment. At the same time, contraction amplitudes for remifentanil (above) and the control (below) are shown. A profound decrease in trabeculae contraction results from ischemia. At the end of 60 min, ischemia oxygen is again applied, and opioid is added to the study probe. The amplitude of contraction is measured several times during reperfusion.

Hypoxia induced a significant, profound decrease of trabeculae contraction amplitude. After 60 min of perfusion with 0% oxygen, 95% argon, and 5% carbon dioxide, the mean amplitude of contraction was 27.7% (SD 12.3%, *p* < 10^–20^) of the baseline contraction.

After the norepinephrine application, contractility increased significantly (compared to the post-hypoxic period), reaching 43.5% of the baseline pre-hypoxic value. During the reoxygenation period, no significant restoration of contractility was observed. The data are given in Table 2.

**Table 2:** Decreased cardiomyocyte contractile function caused by hypoxia and reperfusion. The amplitude of cardiomyocyte contraction is given as % of baseline contraction (100%; NE—norepinephrine application). The number of observations is 208

Baseline	Reperfusion						
	5 min	10 min	15 min	30 min	45 min	60 min	ne
100	27.7 ± 12.3	24.8 ± 14.6	24.5 ± 14.1	27.6 ± 12.3	27.2 ± 13.5	28.1 ± 12.3	43.5 ± 14.1
p vs. 5 min reperfusion	-	ns	ns	ns	ns	ns	0.002

### Effects of remifentanil on the contractile function of ischemic cardiomyocytes

The application of remifentanil improved the cardiomyocyte function compared to the control group. At a 5 μM concentration, cardiomyocytes' contractility was significantly better 30 min from the start of reoxygenation. At a 50 μM concentration, contractility was significantly better 5, 15, 30, 45, and 60 min from the start of reoxygenation.

**Fig. 2:**
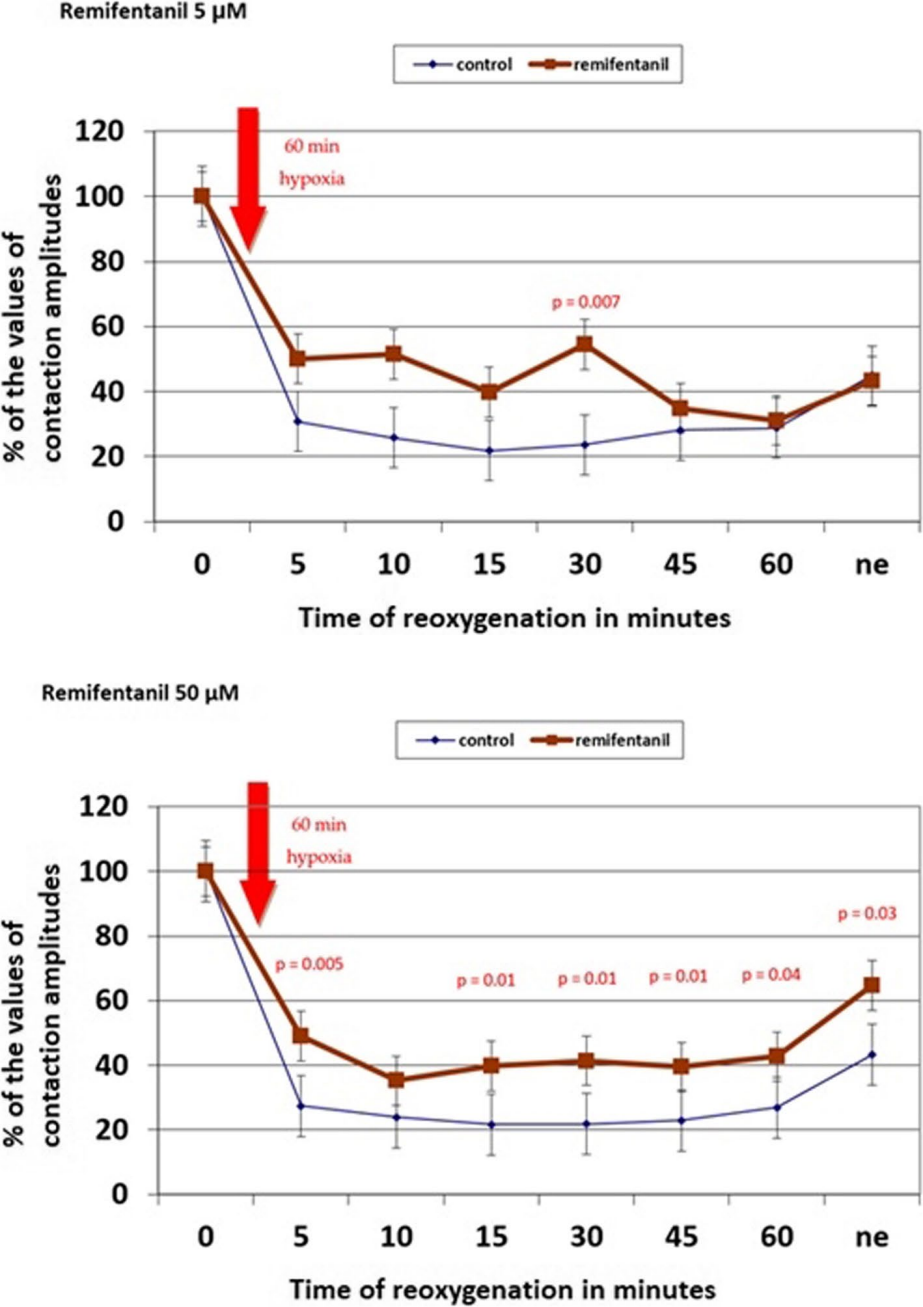
Remifentanil's effects on the contractile function of ischaemic cardiomyocytes. The number of observations: 104. X axis means time of reoxygenation in minutes. Y axis means percentages of the values of contraction amplitudes obtained at the beginning of the experimental protocol. Remifentanil (5 μM or 50 μM) were used from the beginning of the reoxygenation period. Trabecula contractility was assessed as the contraction's maximal amplitude. Measurements were obtained at baseline (Time:0), during reoxygenation (at the 5th, 10th, 15th, 30th, 45th, and 60th minute), and after norepinephrine application (ne)

After norepinephrine application at the end of the experiment, contractility was significantly better in trabeculae perfused with 50 μM of remifentanil. The data are shown in Fig. 2.

### Effects of sufentanil on the contractile function of ischemic cardiomyocytes

**Fig. 3:**
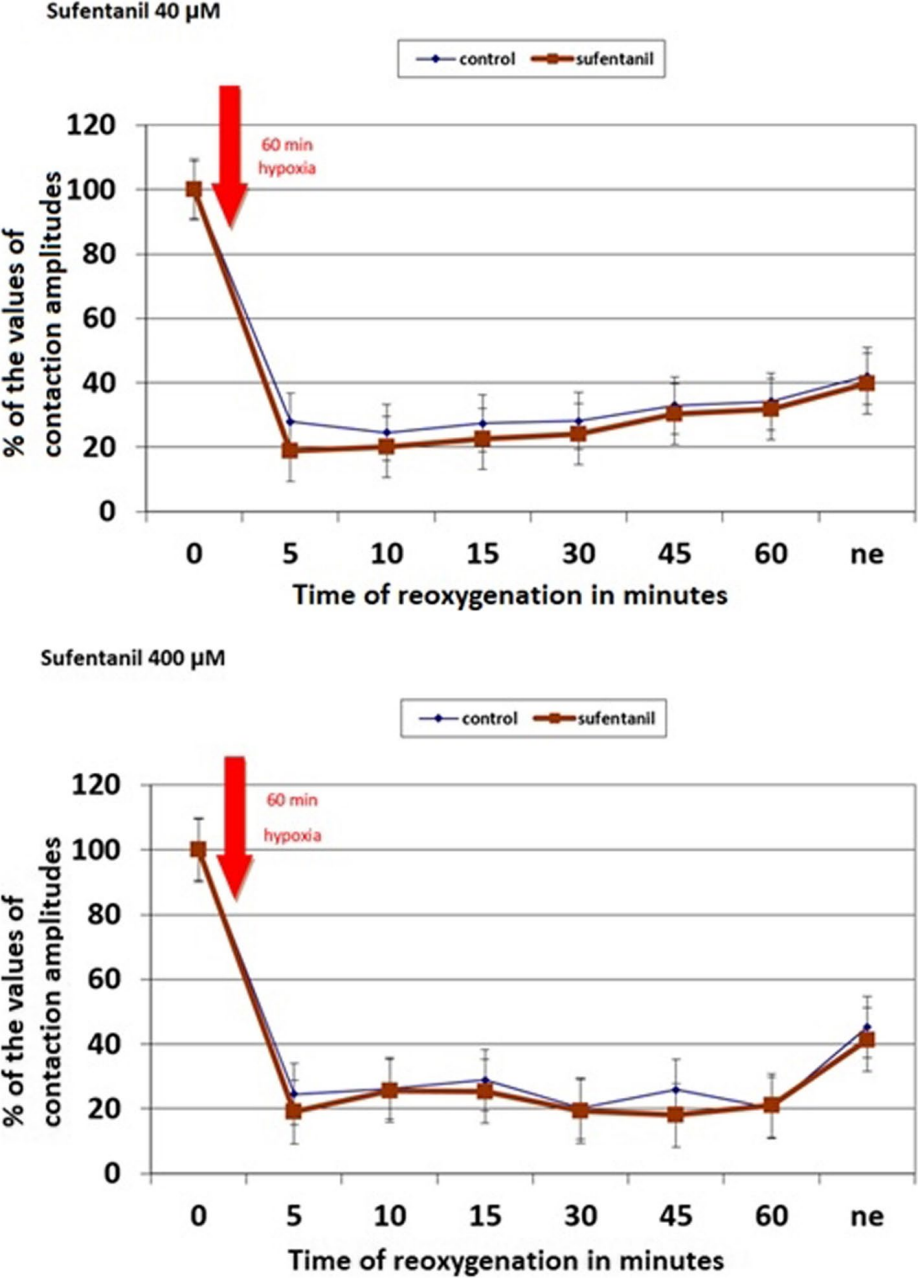
Sufentanil's effects on the contractile function of ischaemic cardiomyocytes. The number of observations: 104. X axis means time of reoxygenation in minutes. Y axis means percentages of the values of contraction amplitudes obtained at the beginning of the experimental protocol. Sufentanil (40 μM or 400 μM) were used from the beginning of the reoxygenation period. Trabecula contractility was assessed as the contraction's maximal amplitude. Measurements were obtained at baseline (Time:0), during reoxygenation (at the 5th, 10th, 15th, 30th, 45th, and 60th minute), and after norepinephrine application (ne)

Sufentanil application did not influence cardiomyocyte function compared to the control group at concentrations of 40 μM and 400 μM. After norepinephrine application at the end of the experiment, contractility was comparable in trabeculae perfused with or without any sufentanil concentration. The data are shown in Fig. 3.

## Discussion

Many studies on opioid conditioning in humans and animals have had encouraging results. Opioids have been investigated as cardioprotective agents against IRI in animal models as well as human in vitro and in vivo studies. Both exogenous and endogenous opioid releasing after nonlethal ischemia protect cardiac and cerebral tissue [19]. Morphine was the first OR agonist proven to confer protection from IRI [20]. Recently, we showed that a high concentration of morphine may confer cardioprotection in human cardiac tissue in vitro [2]. Since excessive opioid doses increase the risk of side effects like respiratory depression, synthetic short-acting opioids have been researched regarding cardioprotective effects due to their selective OR agonism.

Endogenous peptides selectively activate ORs. The OR group consists of three single gene-derived classes: δ-, μ-, and κ-ORs. All these receptors belong to the family of receptors coupled to G-proteins. Crosstalk among other G-protein–coupled receptors like bradykinin, adenosine, and adrenergic receptors may result from receptor peptides' heterodimerization. It has been suggested that several receptor ligands other than opioids may mimic the beneficial effect of the OR stimulation pathway [21].

Most studies have found δ- and κ-, but not μ-receptors expressed in animal cardiomyocytes [8]. Conversely, studies reported evidence of μ-receptors in human cardiac tissues. Using RNA isolation and RT-PCR analysis in human atria and ventricular tissue, Bell and colleagues demonstrated the presence of δ- and μ- receptor in human ventricular tissue at a copy number similar to human atrial tissue, but at a higher copy number than κ-opioid receptors. Although mRNA expression of all three opioid receptors was high in most of the central nervous system area examined, κ- and δ-receptor mRNA expression was detected at very low levels in the heart and μ-receptor mRNA was absent. So origination from neuronal cells in the studied heart tissue could not be excluded. [9–11].

Opioids such as morphine, fentanyl, remifentanil, and sufentanil have varied affinities to ORs, rendering statements regarding subtype involvement in opioid-mediated cardioprotective effect challenging. Remifentanil, an ultra-short-acting opioid rapidly metabolized by blood and tissue esterases [22] with a solid affinity for μ-ORs, is widely used in cardiac anesthesia due to its quick elimination and weak impact on hemodynamics. Most of the studies on the beneficial effect of remifentanil were performed on animal models. Previous research on an animal model have shown that δ- and κ-ORs are involved in cardioprotection, but the role of μ-ORs seems to be marginal because especially in rat cardiomyocytes, only the δ- and κ-ORs are presented, not μ-ORs [12, 13]. It was shown that the beneficial effect of remifentanil preconditioning was abolished by δ- and κ-OR but not by μ-OR antagonists in the isolated rat tissue [23, 24]. Remifentanil is a potent μ-OR agonist and has few effects on δ- and κ-ORs.

Nevertheless, κ-OR stimulation is considered involved in decreasing cardiac IRI related to preconditioning or postconditioning [14, 15]. However, this remains contrary to previous studies that have shown that κ-OR stimulation does not take part in the preconditioning effect [20]. Although the ORs are also found in the neural tissue, it may be questionable that opioid-induced cardioprotection is dependent on processes occurring only in the cardiac tissue. In open-chest anesthetized rat models, the extracardiac μ-OR stimulation may be considered in the cardioprotective remifentanil effect.Moreover, in this model, the beneficial effect was reduced by a selective μ-OR agonist [25]. In turn, a beneficial effect of remifentanil presented in isolated rat hearts precluded a possible influence on the central nervous system [24]. In the rat model of IRI, the significant reduction of cardiac infarct size was observed either in remifentanil preconditioning, postconditioning, and the continuous administration during the ischemic and reoxygenation period [26]. The proposed mechanism of remifentanil action in the animal IRI model reduces oxidative stress [27], increases cell viability, and decreases cell apoptosis [28]. Similarly, in human cardiomyocytes, remifentanil preconditioning confers protection, limiting hypoxia-induced senescence and necroptosis [29].

Sufentanil also predominantly stimulates μ-ORs, and in an animal model similarly presented a cardioprotective effect against IRI. In rats, sufentanil postconditioning reduces infarct size after two hours of reperfusion [25]. Moreover, continuous administration of remifentanil or sufentanil in human heart tissue during the whole pre-ischemic period, ischemia, and reoxygenation confers cardioprotection [26]. These observations suggest a direct cardioprotective effect on cardiomyocytes since neither sufentanil nor remifentanil presented a direct inotropic or lusitropic effect on human heart tissue [25, 30]. Similarly, the reduced infarct size was observed in rats following sufentanil anesthesia administered before, during ischemia, and in the reoxygenation period [31]. In rats, the proposed sufentanil acting mechanism may involve the reduction of oxidative stress and mitochondrial autophagy [32]. Previous studies have shown that δ and κ, but not μ-ORs were present in rat cardiac tissue,and investigators concluded that stimulation of δ and κ-OR conferred cardioprotection. Although, μ-ORs are present in human cardiomyocytes. It cannot be precluded that the presence of μ-ORs has a background in the neural cells. Indeed, cardioprotective effect of μ-OR stimulation in humans, confirmed in the clinical study with remifentanil, strong μ-OR agonist, administered in patients subjected to coronary artery by-pass grafting (CABG) [33]. This results are consistent with our observations. In patients subjected to off-pump CABG, remifentanil administered before sternotomy has been observed to lower troponin I and CK-MB release for twelve to twenty-four hours after the procedure [34]. Moreover, the inotropic support was less required after operation in patients after remifentanil use. The length of time in the intensive care unit (ICU) and the whole hospital stay was shorter in these groups of patients. There is also a higher risk for cardiovascular depression in the early postoperative period [35]. Post-mortem research on opioid users reduced the severity of coronary artery disease [36]. Interestingly, long-term opioid administration may decrease the incidence or extent of cardiac infarction.

Our study's main finding shows the cardioprotective effect of remifentanil, but not sufentanil, on human right-atria cardiomyocytes in in vitro conditions, manifested as the increased amplitude of their contraction during reperfusion after 60 min of ischemia. We presented a remifentanil concentration of 50 μM as the most cardioprotective. Contrary to previous studies, we have been the first to show the effect of remifentanil and sufentanil administered at the end of hypoxia. The beginning of the reoxygenation period presents the model of drug use in acute cardiac infarction. The present study was performed on fragments of isolated human heart tissue. For functional studies, atrial tissue sampling minimalizes the influence of confounding factors, like the effect of drugs or the presence of collateral circulation. We could not assess the infarction size in our research model but tracked the changes in contractility as a functional consequence of cardiac ischemia.

The differences between species in the animal model used in studies might account for the discrepancies in results. In contrast to in vivo studies, isolated cardiac tissue has a limited period of biological stability. The viability of the cardiac tissue differs depending on the animal model. For example, in rats, the application of 30–40 min of ischemia damages 50% of tissue. In pigs, a similar effect is observed after 90 min of ischemia. In our study, due to the limited time of heart tissue stability, we analyzed no more than a 60-min period after reoxygenation. Most studies were performed on young animals. However, intrinsic protective tolerance against IRI may fail with age in humans [37, 38].

Remifentanil mimics cardioprotection via all three ORs. Part of the protective effect may be produced by μ-agonist activity outside the heart. On the other hand, out-of-receptor acting of remifentanil can also bring interesting results. Further studies are needed to explore this unknown acting of sufentanil and remifentanil.

Above mentioned data support an idea on the role of opioids in protecting the human heart against IRI, notwithstanding the controversy concerning the role of OR subtypes. For example, 3, 2, and 7 OR subtypes have been identified for μ-, δ- and κ-OR, respectively [17]. Additionally, receptor crosstalk between ORs complicates the opioid-induced protective reactions. Whether this effect is involved in crosstalk with other ORs or roles of extracardiac µ-ORs remains to be determined.

### Limitations of the study

The results must be interpreted with the limitations ensuing from the methodology. The construction of the experiments assumed a control group obtained from the same patient and the same potentially affecting factors on the final results. We must note that the simulated ischemic model differs from in vivo condition. We used a crystalloid buffer with no transporting system for drugs like peptides. The affinity of remifentanil and sufentanil for OR may vary therefore we may only conclude whether a drug was protective or not. Further discussion about the implicated receptor would require experiments with selective antagonists. We excluded the patients diagnosed with severe valvular heart disease or significant heart failure from the study. We did not excluded male patients only for obtain equal number of females and males. Predominance in our study of older male patients results from high frequency of advanced coronary artery disease required CABG in this group. The presence of co-morbidities and medications may affect the results, but in the in vitro studies it may has minor impact on acute changes of cardiomyocytes’ contractility. The advantage of our study is that we include real patients with atherosclerosis and numerous chronic disorders.

## Conclusions

Remifentanil—but not sufentanil—induces a cardioprotective effect on human right atria cardiomyocytes in in vitro conditions, manifested as increased contraction amplitude during reperfusion after 60 min of ischemia.

## Data Availability

The datasets generated and/or analysed during the current study are available in the Google Drive: https://drive.google.com/drive/folders/1zrzNJyui7STyj44m68IC8s3Xcmj-UeDH

## References

[1] Murry CE, Jennings RB, Reimer KA (1986). Preconditioning with ischemia: a delay of lethal cell injury in ischemic myocardium. Circulation.

[2] Kunecki M, Płazak W, Roleder T, Biernat J, Oleksy T, Podolec P, Gołba KS (2017). ‘Opioidergic postconditioning’ of heart muscle during ischemia/reperfusion injury. Cardiol J.

[3] Granfeldt A, Lefer DJ, Vinten-Johansen J (2009). Protective ischaemia in patients: preconditioning and postconditioning. Cardiovasc Res.

[4] Yellon DM, Alkhulaifi AM, Pugsley WB (1993). Preconditioning the human myocardium. Lancet.

[5] Vinten-Johansen J (2007). Postconditioning: a mechanical maneuver that triggers biological and molecular cardioprotective responses to reperfusion. Heart Fail Rev.

[6] Hausenloy DJ, Candilio L, Evans R, Ariti C, Jenkins DP, Kolvekar S, Knight R, Kunst G, Laing C, Nicholas J, Pepper J, Robertson S, Xenou M, Clayton T, Yellon DM (2015). Remote Ischemic Preconditioning and Outcomes of Cardiac Surgery. N Engl J Med.

[7] Hausenloy DJ, Kharbanda RK, Møller UK, Ramlall M, Aarøe J (2019). Effect of remote ischaemic conditioning on clinical outcomes in patients with acute myocardial infarction (CONDI-2/ERIC-PPCI): a single-blind randomised controlled trial. Lancet.

[8] Ventura C, Bastagli L, Bernardi P, Caldarera CM, Guarnieri C (1989). Opioid receptors in rat cardiac sarcolemma: effect of phenylephrine and isoproterenol. Biochim Biophys Acta.

[9] Tanaka K, Kersten JR, Riess ML (2014). Opioid-induced cardioprotection. Curr Pharm Des.

[10] Headrick JP, See Hoe LE, Du Toit EF, Peart J (2015). Opioid receptors and cardioprotection - ‘opioidergic conditioning’ of the heart. Br J Pharmacol.

[11] Bell SP, Sack MN, Patel A, Opie LH, Yellon DM (2000). Delta opioid receptor stimulation mimics ischemic preconditioning in human heart muscle. J Am Coll Cardiol.

[12] Schultz JJ, Hsu AK, Gross GJ (1997). Ischemic preconditioning and morphine-induced cardioprotection involve the delta (delta)-opioid receptor in the intact rat heart. J Mol Cell Cardiol.

[13] Schultz JE, Hsu AK, Gross GJ (1998). Ischemic preconditioning in the intact rat heart is mediated by delta1- but not mu- or kappa-opioid receptors. Circulation.

[14] Wang GY, Wu S, Pei JM, Yu XC, Wong TM (2001). Kappa- but not delta-opioid receptors mediate effects of ischemic preconditioning on both infarct and arrhythmia in rats. Am J Physiol Heart Circ Physiol.

[15] Wu Y, Wan J, Zhen WZ, Chen LF, Zhan J, Ke JJ, Zhang ZZ, Wang YL (2014). The effect of butorphanol postconditioning on myocardial ischaemia reperfusion injury in rats. Interact Cardiovasc Thorac Surg.

[16] Zhao M, Joo DT (2008). Enhancement of spinal N-methyl-D-aspartate receptor function by remifentanil action at delta-opioid receptors as a mechanism for acute opioid induced hyperalgesia and tolerance. Anesthesiology.

[17] Dietis N, Rowbotham DJ, Lambert DG (2011). Opioid receptor subtypes: fact or artifact?. Br J Anaesth.

[18] Wu Y, Gu EW, Zhu Y, Zhang L, Liu XQ, Fang WP (2012). Sufentanil limits the myocardial infarct size by preservation of the phosphorylated connexin 43. Int Immunopharmacol.

[19] Arabian M, Aboutaleb N, Soleimani M, Ajami M, Habibey R, Rezaei Y, Pazoki-Toroudi H (2018). Preconditioning with morphine protec ts hippocampal Ca1 neurons from ischemia-reperfusion injury via activation of the mTOR pathway. Can. J Physiol. Pharmacol.

[20] Schultz JE, Rose E, Yao Z, Gross GJ (1995). Evidence for involvement of opioid receptors preconditioning in rat hearts. Am J Physiol.

[21] Gupta A, Mulder J, Gomes I, Rozenfeld R, Bushlin I, Ong E, Lim M, Maillet E, Junek M, Cahill CM, Harkan T, Devi LA (2010). Increased abundance of opioid receptor heteromers after chronic morphine administration. Sci Signal.

[22] Dershwitz M, Randel GI, Rosow CE, Fragen RJ, Connors PM, Librojo ES, Shaw DL, Peng AW, Jamerson BD (1995). Initial clinical experience with remifentanil, a new opioid metabolized by esterases. Anest Analg.

[23] Zhang Y, Irwin MG, Wong TM (2004). Remifentanil preconditioning protects against ischemic injury in the intact rat heart. Anesthesiology.

[24] Zhang Y, Irwin MG, Wong TM, Chen M, Cao CM (2005). Remifentanil preconditioning confers cardioprotection via cardiac kappa- and delta-opioid receptors. Anesthesiology.

[25] Hanouz JL, Yvon A, Guesne GG, Eustratiades C, Babatasi G, Rouet R, Ducouret P, Khayat A, Bricard H, Gerard JL (2001). The In Vitro Effects of Remifentanil, Sufentanil, Fentanyl, and Alfentanil on Isolated Human Right Atria. Anesth Analg.

[26] Lemoine S, Zhu L, Massetti M, Gérard J-L, Hanouz J-L (2011). Continuous administration of remifentanil and sufentanil induces cardioprotection in human myocardium, in vitro. Acta Anaesthesiol Scand.

[27] Zhou Q, Song J, Wang Y, Lin T (2020). Remifentanil attenuates cardiac dysfunction, lipid peroxidation and immune disorder in rats with isoproterenol-induced myocardial injury via JNK/NF-KB p65 inhibition. Ann Transl Med.

[28] Cheng L, Wu Y, Tang J, Zhang C, Cheng H, Jiang Q, Jian C (2021). Remifentanil protects against myocardial ischemia/reperfusion injury via miR-205-mediated regulation of PINK. J Toxicol Sci.

[29] Lewinska A, Adamczyk-Grochala J, Bloniarz D, Horeczy B, Zurek S, Kurowicki A, Woloszczuk-Gebicka B, Widenka K, Wnuk M (2020). Remifentanil preconditioning protects against hypoxia-induced senescence and necroptosis in human cardiac myocytes in vitro.

[30] Duman A,  Sahin AS, Atalik KE, Ogün C, Ulusoy HB, Durgut K, Okesli S (2003). The in vitro effects of remifentanil and fentanyl on isolated human right atria and saphenous veins. J Cardiothorac Vasc Anesth.

[31] Ter Horst EN, Krijnen PAJ, Flecknell P, Meyer KW, Kramer K, van der Laan AM, Piek JJ, Niessen HWM (2018). Sufentanil-medetomidine anaesthesia compared with fentanyl/fluanisone-midazolam is associated with fewer ventricular arrhythmias and death during experimental myocardial infarction in rats and limits infarct size following reperfusion. Lab Anim.

[32] Wu Q, Shang Y, Bai Y, Wu Y, Wang H, Shen T (2021). Sufentanil preconditioning protects against myocardial ischemia/reperfusion injury via miR-125a/DRAM2 axis. Cell Cycle.

[33] Wong GTC, Huang Z, Ji S, Irwin MG (2010). Remifentanil reduces the release of biochemical markers of myocardial damage after coronary artery bypass surgery: a randomized trial. J Cardiothorac Vasc Anesth.

[34] Xu ZD, Jin M, He WX, Xia SX, Zhao YF, He B, Cao DX, Peng SL, Li J, Cao MH (2009). Remifentanil preconditioning lowers cardiac troponin I levels in patients undergoing off-pump coronary artery by-pass graft surgery. Nan Fang Yi Ke Da Xue Xue Bao..

[35] Pleym H, Stenseth R, Wiseth R, Karevold A, Dale O (2004). Supplemental remifentanil during coronary artery bypass grafting is followed by a transient postoperative cardiac depression. Acta Anaesthesiol Scand.

[36] Marmor M, Penn A, Widmer K, Levin RI, Maslansky R (2004). Coronary artery disease and opioid use. Am J Cardiol.

[37] Peart JN, Pepe S, Reichelt ME, Beckett N, See Hoe L, Ozberk V, Niesman IR, Patel HH, Headrick JP (2014). Dysfunctional survivalsignaling and stress-intolerance in aged murine and human myocardium. Exp Gerontol.

[38] Chun KJ, Park YH, Kim JS, Jang Y, Kim JH, Kim J, Lee MY (2011). Comparison of 5 different remifentanil strategies against myocardial ischemia-reperfusion injury. J Cardiothorac Vasc Anesth.

